# Ferroptosis and pyroptosis signatures in critical COVID-19 patients

**DOI:** 10.1038/s41418-023-01204-2

**Published:** 2023-08-15

**Authors:** Cédric Peleman, Samya Van Coillie, Symen Ligthart, Sze Men Choi, Jan De Waele, Pieter Depuydt, Dominique Benoit, Hannah Schaubroeck, Sven M. Francque, Karolien Dams, Rita Jacobs, Dominique Robert, Ria Roelandt, Ruth Seurinck, Yvan Saeys, Mohan Rajapurkar, Philippe G. Jorens, Eric Hoste, Tom Vanden Berghe

**Affiliations:** 1 Laboratory of Experimental Medicine and Paediatrics, Infla-Med Centre of Excellence, University of Antwerp, Antwerp, Belgium; 2 Department of Gastroenterology and Hepatology, Antwerp University Hospital, Edegem, Belgium; 3 VIB-UGent Center for Inflammation Research, Ghent, Belgium; 4 Department of Biomedical Molecular Biology, Ghent University, Ghent, Belgium; 5 Division of Intensive Care, Medicine, Antwerp University Hospital, Edegem, Belgium; 6 Intensive Care Unit, Department of Internal Medicine and Pediatrics, Ghent University Hospital, Ghent University, Ghent, Belgium; 7 Department of Applied Mathematics, Computer Science and Statistics, Ghent University, Ghent, Belgium; 8 Department of Nephrology, Muljibhai Patel Society for Research in Nephro-Urology, Nadiad, India; 9 Laboratory of Pathophysiology, Department of Biomedical Sciences, University of Antwerp, Antwerp, Belgium

**Keywords:** Biomarkers, Interleukins, Infectious diseases

## Abstract

Critical COVID-19 patients admitted to the intensive care unit (ICU) frequently suffer from severe multiple organ dysfunction with underlying widespread cell death. Ferroptosis and pyroptosis are two detrimental forms of regulated cell death that could constitute new therapeutic targets. We enrolled 120 critical COVID-19 patients in a two-center prospective cohort study to monitor systemic markers of ferroptosis, iron dyshomeostasis, pyroptosis, pneumocyte cell death and cell damage on the first three consecutive days after ICU admission. Plasma of 20 post-operative ICU patients (PO) and 39 healthy controls (HC) without organ failure served as controls. Subsets of COVID-19 patients displayed increases in individual biomarkers compared to controls. Unsupervised clustering was used to discern latent clusters of COVID-19 patients based on biomarker profiles. Pyroptosis-related interleukin-18 accompanied by high pneumocyte cell death was independently associated with higher odds at mechanical ventilation, while the subgroup with high interleuking-1 beta (but limited pneumocyte cell death) displayed reduced odds at mechanical ventilation and lower mortality hazard. Meanwhile, iron dyshomeostasis with a tendency towards higher ferroptosis marker malondialdehyde had no association with outcome, except for the small subset of patients with very high catalytic iron independently associated with reduced survival. Forty percent of patients did not have a clear signature of the cell death mechanisms studied in this cohort. Moreover, repeated moderate levels of soluble receptor of advanced glycation end products and growth differentiation factor 15 during the first three days after ICU admission are independently associated with adverse clinical outcome compared to sustained lower levels. Altogether, the data point towards distinct subgroups in this cohort of critical COVID-19 patients with different systemic signatures of pyroptosis, iron dyshomeostasis, ferroptosis or pneumocyte cell death markers that have different outcomes in ICU. The distinct groups may allow ‘personalized’ treatment allocation in critical COVID-19 based on systemic biomarker profiles.

## Introduction

Infection with the severe acute respiratory syndrome coronavirus-2 (SARS-CoV-2) may cause coronavirus disease 2019 (COVID-19) which manifests as severe pneumonitis with further deterioration into an acute respiratory distress syndrome (ARDS) but also multiple organ dysfunction [1, 2]. COVID-19 developing into a global pandemic caused an unprecedented surge in intensive care unit (ICU) admissions [3, 4]. Critical COVID-19 is not only associated with a high ICU mortality rate but also long-term effects [5–10]. These observations motivate the search for insight in the underlying pathophysiology of COVID-19 and treatment options for critical COVID-19 patients.

Cell death mediated tissue damage in critical COVID-19 likely results from an overactive, dysregulated immune response and vascular “disease”, rather than direct virus-mediated damage [11]. In general, cell death with the release of cellular content may promote necroinflammation leading to clinical deterioration and death in the ICU patient with organ dysfunction [12]. Ferroptosis is a form of regulated necrosis executed by iron-catalysed peroxidation of polyunsaturated fatty acids in membrane phospholipids, termed lipid peroxidation (LPO). Non-transferrin, non-ferritin bound iron, also called catalytic iron (Fe_c_), can promote this process via Fenton reactions [13]. Breakdown products of LPO, such as malondialdehyde (MDA), serve as markers of ferroptosis [14]. Systemic levels of Fe_c_ can be assessed, along with proteins related to iron homeostasis including ferritin, lactoferrin and myoglobin. Due to a dysregulation of iron homeostasis, the presence of ischemia-reperfusion and reactive oxygen species in severe COVID-19, ferroptosis has been hypothesized to be a major, druggable detrimental factor [15–17]. Indeed, ferroptosis can be inhibited by lipophilic radical trapping antioxidants [14, 18–20]. We recently showed the lifesaving capacity of a highly soluble third generation lead ferroptosis inhibitor in experimental multiorgan dysfunction [21]. Pyroptosis, another type of cell death executed by pore formation by gasdermin D N-terminal ends, may also play a role in acute lung injury due to COVID-19 [22–24]. The pores facilitate the release of interleukin-1 beta (IL-1β) and interleukin-18 (IL-18) in the environment. Based on these upregulated pyroptosis markers in peripheral blood mononuclear cells (PBMCs) from COVID-19 patients, pyroptosis and its interleukins are studied too as possible therapeutic targets in critical COVID-19 patients [25–27]. We have previously shown that simultaneous neutralisation of IL-1 and IL-18 is lifesaving in experimental sepsis models [28].

In view of regulated cell death as a potential and promising target in critical COVID-19, we monitored ferroptosis, iron dysregulation and pyroptosis signals in critical COVID-19 patients, early after ICU admission. As COVID-19 presents with pulmonary damage, we also measured soluble receptor for advanced glycation end products (sRAGE) as a marker for alveolar type I pneumocyte cell damage [29, 30]. In addition, we examined levels of inflammation-induced growth and differentiation factor 15 (GDF15) protein, which is secreted in case of local tissue damage and regulates iron metabolism [31–33]. Lastly, we investigated the association between these biomarkers and disease severity markers, mechanical ventilation, and mortality.

## Subjects and methods

### Study populations

From April 1st 2020 to April 27th 2021, 120 critical COVID-19 patients were included in the cohort study upon ICU admission (100 patients at Ghent University Hospital (UZGhent) and 20 patients at Antwerp University Hospital (UZA)). Identical criteria for ICU admission in the two centers were severe hypoxemic respiratory failure or deterioration of hemodynamic status. Inclusion criteria for the study were age of 18 years or older, admitted to the ICU with PCR proven COVID-19, with an arterial line for blood sampling, and informed consent by the patient or their closest relatives. Plasma and serum samples were collected on the first three consecutive days of ICU stay, with the first day of sampling equaling the day of ICU admission. This timeframe was chosen since disease severity early in ICU stay has been proposed to be predictive for final outcome [34]. Samples were stored at –80 °C. Plasma samples from 20 adult non-septic post-operative ICU patients (PO) who had underwent major intracranial surgery (resection of a cerebral tumor or clipping of an aneurysm) admitted at the ICU of UZA for postoperative monitoring (Amendment 17/10/119, reference B300201732219) and reported elsewhere [35], were analysed as well. Furthermore, samples from 39 gender-matched healthy adult volunteers served as healthy controls (HC). All protocols were conformed to the ethical guidelines of the latest version of the Declaration of Helsinki. The study was approved by the Ethical Committees of UZGhent (reference BC-07568) and UZA (20/14/169, Edge nr. 001072, ref. B3002020000057).

### Markers of ferroptosis, iron metabolism, pyroptosis and cell damage/cell death

Ferroptosis was assessed by the biomarker plasma MDA and catalytic iron (Fe_c_). The latter together with ferritin and lactoferrin reflects iron metabolism. Myoglobin release could contribute to increased catalytic iron and is therefore considered as iron metabolism indicator. Pyroptosis-related interleukins IL-1β and IL-18 were assessed in the plasma. sRAGE and GDF15 were measured as markers for alveolar type I pneumocytes damage and cell damage. In addition, levels of interleukin-6 (IL-6) were measured in plasma, since this biomarker is known to bear prognostic importance in COVID-19. Plasma MDA, reflecting total lipid peroxidation in the body, was measured using the N-methyl-2-phenylindole colorometric assay, as described elsewhere [21, 36]. Briefly, 50 µL of the test sample was added to a reagent mixture containing N-methyl 2-phenylindole, acetonitrile and methanol. At optimal temperature and pH, MDA from the sample combines with N-methyl 2-phenylindole to form a chromogen. By comparison with a standard curve the total amount of MDA (in µM) in test sample was determined. Catalytic iron (Fe_c_) (expressed in µmol/L) was measured in plasma using a modified version of the bleomycin detectable iron assay [37]. As bleomycin degrades DNA in the presence of catalytic iron, a thiobarbituric acid reactive substance is formed. The latter reacts with thiobarbituric acid to form a chromogen whose intensity is measured at 532 nm using a spectrophotometer. All reagents were treated beforehand with Chelex 100 (Bio-Rad; #1421253), except for bleomycin, to avoid iron contamination. Ferritin levels in serum samples were measured on fresh samples in the clinical laboratories of the participating hospitals using standard laboratory techniques. Lactoferrin, myoglobin, IL-1β, IL-18, sRAGE and GDF15, as well as IL-6, were measured in plasma (as pg/mL) in a central lab using validated bead-based multiplex immune assays on a Luminex 200 instrument (Luminex Corporate, Austin, TX, US). Measurements were performed with blinding for patient outcomes and group membership.

### Outcomes

Clinical outcomes included 90-day mortality after ICU admission and the use of invasive mechanical ventilation in ICU. Data concerning the Sequential Organ Failure Assessment (SOFA) score [38] (a validated score for organ failure), the Acute Physiology and Chronic Health Evaluation (APACHE) II score [39] (a validated score for severity of critical illness scored after 24 h in ICU), partial pressure of oxygen to fraction of inspired oxygen (P/F ratio) (a marker for lung injury), ventilation-free days (VFDs) and 90-day mortality were extracted from the electronic patient database management system. VFDs were defined as: 28 minus “x” if successfully weaned from mechanical ventilation at “x” days after ICU admission; in case of death within 28 days of mechanical ventilation or continued mechanical ventilation for more than 28 days, the number of VFDs is zero [40]. Of note, 16 out of 120 COVID-19 patients were also included in an open-label clinical trial and received immunomodulating therapy (anakinra, siltuximab, tocilizumab or standard-of-care) shortly before or upon ICU admission; this trial was published previously and yielded negative results [41]. As a sensitivity analysis, we repeated the correlation analysis without the subset of COVID-19 patients enrolled in the immunomodulatory trial.

### Unsupervised clustering of critical COVID-19 patients

COVID-19 patients were separated into latent subgroups/clusters based on profiles of all biomarkers by Gaussian Mixture modeling. Moreover, unsupervised clustering of COVID-19 patients was performed based on trajectories/kinetics of individual biomarkers over the three consecutive measurements using longitudinal k-means clustering. Details of the procedures can be found in Supplementary Information. All R coding for the unsupervised clustering can be found at: https://github.com/cedricpeleman.

### Statistical analyses

Continuous variables were tested for normal distribution and presented as mean and standard deviation (SD) or median and interquartile range (IQR), whereas percentages and ratios were used for categorical variables. 95% confidence intervals are mentioned where possible. Biomarkers were log2 transformed and the maximum value of all measurements during the first three days after ICU admission was used, unless otherwise specified. Differences in baseline characteristics and biomarkers of the three groups of interest were assessed using one-way ANOVA in case of normal distribution and Kruskal-Wallis test for non-normality data; pairwise comparisons were performed using the Wilcoxon-rank sum test. Associations among biomarkers and between biomarkers and disease severity scores were investigated using a Spearman’s rank correlation coefficient. Correlation coefficients were interpreted as follows: 0–0.1 corresponds to negligible correlation, 0.1–0.39 corresponds to weak correlation, 0.4–0.69 corresponds to moderate correlation, 0.7–0.89 corresponds to strong agreement, and 0.9 or higher indicates very strong correlation [42]. *T* tests were run to test the null hypothesis that the correlation coefficient is zero. Benjamini-Hochberg correction for multiple hypothesis testing was applied when appropriate [43]. We further performed logistic and linear regression and Cox proportional hazards models to analyse the association between cluster membership and 90-day mortality, VFDs and need of mechanical ventilation. Statistical analyses were performed in R version 4.1.1 using packages ggstatsplot and survival [44]. All figures were generated in RStudio.

## Results

### Patient population and demographic characteristics

The characteristics of the critical COVID-19 patients, hereafter termed ‘COVID-19’ group, healthy controls (HC) and post-operative ICU controls (PO) are summarised in Table 1. COVID-19 patients were older and had a higher body mass index (BMI) compared to the other groups (Supplementary Fig. S1). Use of corticosteroids in this cohort of COVID-19 patients was limited (<7%) on all sampling days. COVID-19 patients displayed more arterial hypertension (45%) and more type 2 diabetes mellitus (25%) compared to HC (Supplementary Table S1).

**Table 1:** Demographics of study population.

Characteristics	COVID-19 (*n* = 120)	HC (*n* = 39)	PC (*n* = 20)	Total cohort	*p* value
Age, years					<0.001
Median (IQR)	64 (14)	50 (11.75)	52 (20.25)	59 (18)	
Min-max range	24–81	25-64	31-66	24-81	
Gender, n (%)					0.269
Female	39 (32.5)	18 (47.37)	11 (55)	68 (38.2)	
Male	81 (67.5)	20 (52.63)	9 (45)	110 (61.80)	
Body mass index (BMI), kg/m²					<0.001
Median (IQR)	28.37 (7.72)	24.17 (3.39)	24.81 (2.77)	26.4 (6.37)	
Min-max range	15.43–51.90	17.78–29.41	20.11–30.43	15.43–51.90	

Highest Sequential Organ Failure Assesment (SOFA) during first three days in ICU			
median (IQR)	9 (9)	NA	NA
Min-max range	2-18	NA	NA
Acute Physiology and Chronic Health Evaluation (APACHE) II score			
median (IQR)	18.5 (13)	NA	NA
Min-max range	2-40	NA	NA
Mechanical ventilation during ICU hospitalisation			
n (%)	79 (65.83)	NA	NA
Corticosteroid use first three days in ICU			
first day, n (%)	4 (3.33)	NA	NA
second day, n (%)	5 (4.17)	NA	NA
third day, n (%)	8 (6.67)	NA	NA
Vassopression need during first three days in ICU			
first day, n (%)	41 (34.17)	NA	NA
second day, n (%)	43 (35.83)	NA	NA
third day, n (%)	45 (37.5)	NA	NA

Demographics of the study populations of critical COVID-19 patients (COVID-19), healthy controls (HC) and post-operative ICU controls (PC). Data presented as median (IQR) for continuous variables or percentage for numbers. Not applicable (NA).

### Critical COVID-19 patients show increased biomarkers of ferroptosis, iron dyshomeostasis, pyroptosis and cell death early in ICU stay

**Fig. 1:**
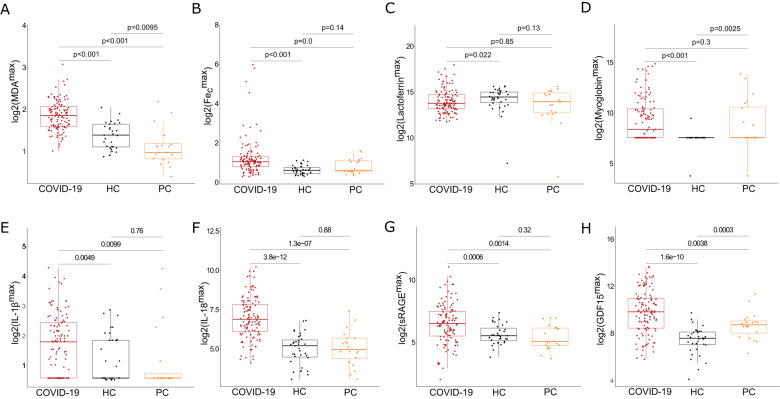
Comparison of cell death biomarkers in critical COVID-19 patients, healthy controls and post-operative intensive care unit controls. **A**-**D** Boxplots representing log2-transformed values of ferroptosis marker malondialdehyde (MDA) and iron homeostasis markers catalytic iron (Fe_c_), lactoferrin and myoglobin, as measured in plasma. The highest value of each biomarker in the first three days after intensive care unit (ICU) admission is presented for COVID-19 patients (COVID-19). These values were compared with single measurements in healthy controls (HC) and post-intracranial surgery non-COVID-19 ICU controls (PC). **E**-**F** Likewise, log2-transformed values of pyroptosis related interleukins, interleukin-1 beta (IL-1β) and interleukin-18 (IL18), are presented for the three groups. **G**-**H** Systemic levels of soluble receptor for advanced glycation end products (sRAGE), reflecting alveolar pneumocyte damage, and growth differentiation factor 15 (GDF15) are presented for the three groups. The latter reflect inflammation-induced tissue damage and has an immunomodulatory role. Pairwise comparisons of values of biomarkers were performed using the Wilcoxon-rank sum test.

The highest levels of each biomarker during the first three days after ICU admission of the COVID-19 group were compared with the levels of the other study groups. Descriptive data of these biomarkers can be found in a Supplementary Table S2. The mean value of MDA was higher in COVID-19 patients compared to HC and PO (Fig. 1A). Plasma levels of iron homeostasis markers Fe_c_ and myoglobin were higher in COVID-19 compared to HC (but not different from PO), while lactoferrin was lower in COVID-19 compared to HC (Fig. 1B–D). Mean plasma levels of IL-1β and IL-18, as products of inflammasome activity and pyroptosis, were higher in COVID-19 than in the other groups (Fig. 1E, F), as was the case for the alveolar cell damage marker sRAGE and GDF15 in the COVID-19 group (Fig. 1G, H). Supplementary Fig. S2 shows increased plasma IL-6 levels in COVID-19 compared to controls. Given the variation in biomarker levels in COVID-19 patients, the percentage of patients with increased or decreased biomarker levels (and combinations thereof) was calculated, using the 95% confidence interval of each biomarker in HC as reference interval (Supplementary Table 3). Subsets of patients displayed increased levels of MDA and Fe_c_ (19.17%), or combined increase in IL-1β and IL-18 (6.67%) (Supplementary Table 4). Thus, subsets of COVID-19 patients show a systemic signature of ferroptosis, iron dysregulation or pyroptosis during first days of ICU admission.

### Specific correlations between biomarkers of cell death and disease severity scores early in ICU stay

**Fig. 2:**
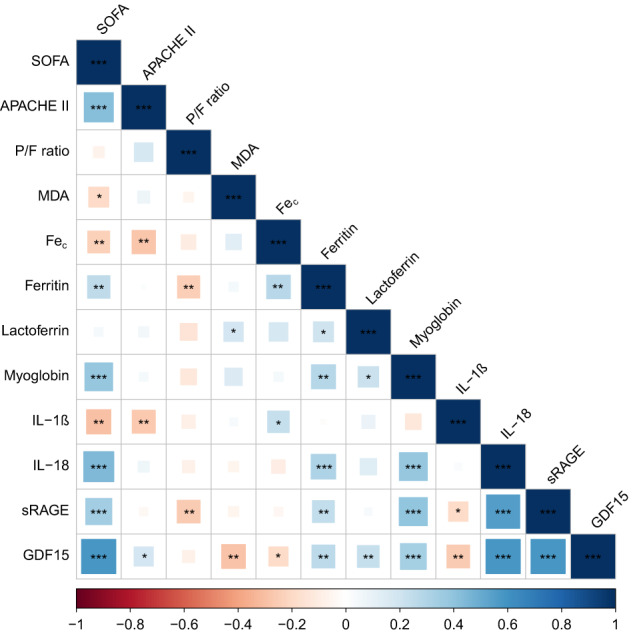
Correlogram summarising associations between biomarkers and disease severity scores in the first three days after ICU admission of critical COVID-19 patients. We explored associations between the highest value of biomarkers for ferroptosis (i.e. MDA), iron dyshomeostasis (catalytic iron [Fe_c_], ferritin, lactoferrin, myoglobin), pyroptosis-related interleukins (interleukin-1 beta [IL-1β] and interleukin-18 [IL-18]), soluble receptor of advanced glycation end products (sRAGE) and growth differentiation factor 15 (GDF15), as measured systemically within the first three consecutive days after ICU admission. In addition, these biomarkers were compared with the highest Sequential organ failure assessment (SOFA) score, APACHE II score (on first day) and lowest P/F ratio in the same time period early during ICU stay. Spearman’s correlation coefficients between two biomarkers and/or clinical scores are presented at the intersection of columns and rows in lower left-hand corner of the figure. *T* tests were performed to assess significance of correlation coefficients; *p < 0.05, **p < 0.01, ***p < 0.001.

We examined correlations between biomarkers of cell death and disease severity scores early in the ICU stay (Fig. 2). The ferroptosis marker MDA was weakly correlated with lactoferrin, but no notable correlations were found with other markers of iron dysregulation. However, we observed correlations among markers of altered iron homeostasis (ferritin correlated weakly with Fe_c_, lactoferrin and myoglobin). Furthermore, the highest values of IL-1β and IL-18 showed no correlation, but IL-1β correlated weakly with Fe_c_, while IL-18 showed an association with ferritin and myoglobin. Plasma levels of sRAGE correlated weakly with ferritin, myoglobin and IL-1β, while GDF15 correlated weakly with all other biomarkers. Of note, both sRAGE and GDF15 correlated moderately with IL-18. Secondly, we assessed the relation between biomarkers and worst clinical scores during first 3 days of ICU admission. The highest SOFA score displayed weak negative correlations with MDA and Fe_c_ but weak positive correlation with ferritin, myoglobin, IL-1β and sRAGE, as well as a moderate correlation with IL-18 and GDF15. The APACHEII score negatively correlated with Fe_c_ and IL-1β. Lastly, the lowest P/F ratio correlated negatively with ferritin and sRAGE (Fig. 2). As a sensitivity analysis, we explored these correlations in the subset of COVID-19 patients not enrolled in the immunomodulatory trial (*n* = 106) and found the same associations (Supplementary Fig. S3).

### Unsupervised clustering of COVID-19 reveals subgroups with adverse clinical outcome

**Fig. 3:**
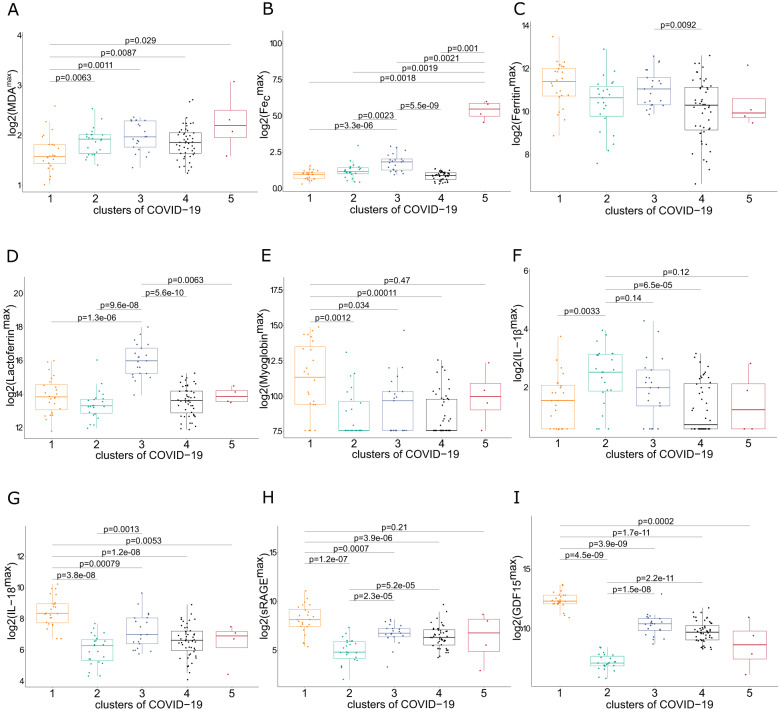
Comparison of biomarkers early in ICU stay between biomarker-based clusters of critical COVID-19 patients. **A**–**I** Log2-transformed values of ferroptosis, iron dysregulation, pyroptosis-related interleukins, sRAGE and GDF15 (highest values during first three days after ICU admission) are presented with boxplots (mean, interquartile range and min-max values) in patients from five biomarker-based clusters (1, 2, 3, 4, 5). Pairwise comparisons of values of biomarkers were performed using the Wilcoxon-rank sum test.

**Fig. 4:**
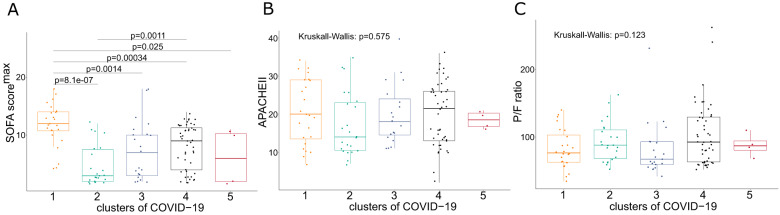
Comparison of disease severity scores in the first three days after ICU admission with biomarker-based clusters of critical COVID-19 patients. **A** Boxplot represents the mean, interquartile range and min-max values of the highest Sequential organ failure assessment (SOFA) score in different clusters of critical COVID-19 patients, as defined by Gaussian Mixture modeling based on biomarker profiles. Similarly, APACHE II score (on first day) (**B**) and lowest P/F ratio in the first three days after ICU admission (**C**) were plotted among different biomarker-based clusters of critical COVID-19 patients. In case of significant differences in mean values of these disease severity scores among the five clusters on Kruskal-Wallis test, pairwise comparisons were performed using the Wilcoxon-rank sum test.

Unsupervised machine learning was used to separate COVID-19 patients based on their biomarker levels (including ferritinemia) into clusters or subgroups with distinct biomarkers profiles. Separation of all COVID-19 patients (*n* = 120) into five clusters was most appropriate, as explained in more detail in supplementary information (Supplementary Fig. S4). Next, we compared mean values of all biomarkers among those five clusters to identify cluster-defining traits (Fig. 3A-I). Cluster 1 (*n* = 24) displayed low MDA, but the highest levels of myoglobin, IL-18, sRAGE and GDF15. Clusters 2 (*n* = 23) and 3 (*n* = 21) represent COVID-19 patients with the highest IL-1β (with low sRAGE and GDF15) and lactoferrin (and high Fe_c_ and GDF15), respectively. Cluster 4 (*n* = 48) has no defining biomarker profile, while the cluster 5 consisted of a small number of patients (*n* = 4) with very high levels of MDA, Fe_c_ and myglobin. Considering the biomarker reference values in HC (Supplementary Table S5), cluster 1 and 2 both have a high pyroptosis-related interleukin, but high and low pneumocyte cell death, respectively. Cluster 3 represents COVID-19 patients with iron dyshomeostasis and a tendency toward highest MDA, while cluster 5 has a ferroptotic signature. Forty percent of patients (cluster 4) have no defining biomarker among the ones measured in this study. Cluster 1 displayed higher SOFA levels during the first three days after ICU admission compared to all other clusters (Fig. 4A). No difference was found in APACHEII or lowest P/F ratio among the clusters (Fig. 4B, C).

### Specific correlations between cluster membership and clinical outcomes

**Fig. 5:**
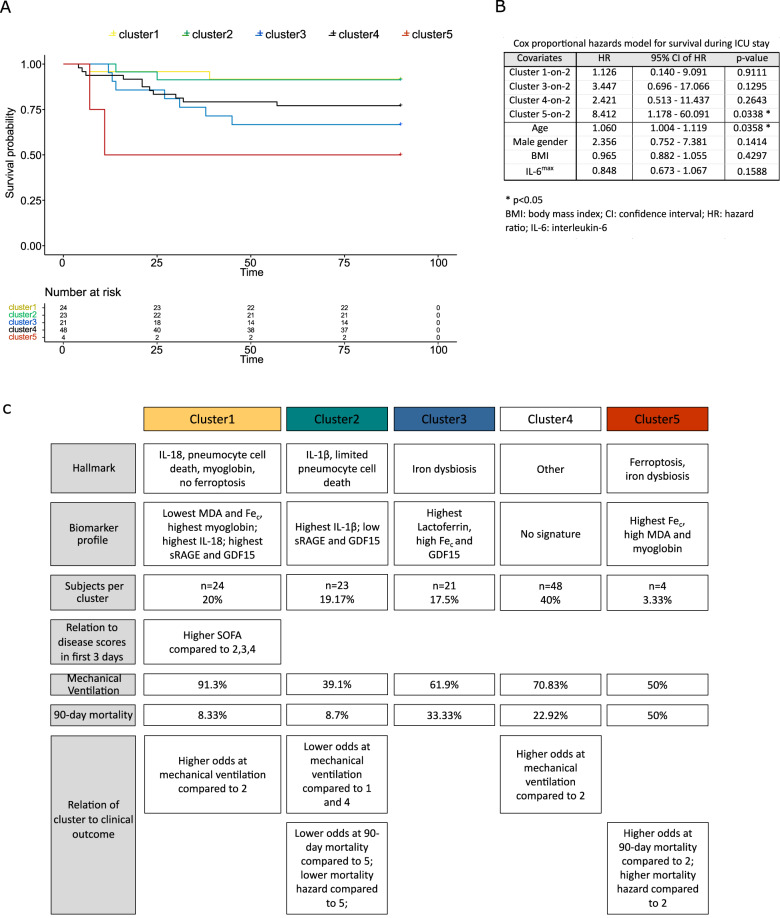
Survival analysis among biomarker-based clusters of critical COVID-19 patients. **A** Survival curves of each of the five biomarker-based clusters of critical COVID-19 patients (as defined by Gaussian Mixture modeling) were plotted using the Kaplan-Meier method. **B** Next, Cox proportional hazards modeling was used to assess the independent effect of cluster membership on the hazard of mortality, thereby adjusting for age, gender, body mass index and levels of interleukin-6. Hazard ratios (HR), 95% confidence intervals of HRs and *p* values were calculated for dummy variables which compare each biomarker-based cluster with the reference cluster 2. Cluster 5 comprises patients with very high MDA and Fe_c_ and independently associated with a higher hazard ratio of mortality compared to cluster 2 in multivariate analysis. **C** Schematic summary for each biomarker-based cluster: cluster name, the biomarker profile, the number of COVID-19 patients per cluster, relation of each cluster to disease score in the first 3 days after ICU admission, association between ventilation and survival outcome parameters and cluster membership.

We examined possible relations of cluster membership with clinical outcomes through regression analysis, thereby correcting for age, gender, BMI and interleukin-6 (IL-6) levels, which are reported to relate to outcome in COVID-19. The ‘high IL-1β’-cluster 2 (without pneumocyte cell death) was associated with lower risk of mechanical ventilation, in contrast to the “high IL-18”-cluster 1 (with pneumocyte cell death) and “undefined”-cluster 4 (Table 2). None of the clusters were associated with the number of ventilation-free days (VFDs) (Supplementary Fig. S5). Cluster 5 with ferroptotic signature had higher probability of 90-day mortality than ‘high IL-1β’-cluster 2 (Table 3). Age was also associated with higher 90-day mortality (Table 3). The “ferroptotic”-cluster 5 showed the lowest survival rates on Kaplan-Meier survival curves and displayed higher hazards ratios of mortality compared to cluster 2 after adjusting for the covariates (Fig. 5A, B). Figure 5C summarizes the findings concerning cluster-defining traits and their relationship with clinical outcomes.

**Table 2:** Association between mechanical ventilation in ICU and biomarker-based clusters in critical COVID-19 patients.

Need of mechanical ventilation during ICU admission in critical COVID-19 patients						
	Clusters	1	2	3	4	5
Need of mechanical ventilation	yes	21	9	13	34	2
	no	2	14	8	14	2
	Percentage of COVID-19 with need of mechanical ventilation per cluster (%)	91.3	39.1	61.9	70.83	50

Multivariate logistic regression for biomarker-based clusters associated with Mechanical ventilation during ICU stay			
Covariates	OR	95% CI of OR	*p* value
Intercept	0.101	0.002–4.614	0.240
Cluster 1-on-2	8.381	1.351–51.975	0.0224 *
Cluster 3-on-2	1.886	0.519–6.857	0.336
Cluster 4-on-2	3.416	1.160–10.056	0.0258 *
Cluster 5-on-2	1.484	0.158–13.920	0.730
Age	0.996	0.959–1.035	0.856
Gender male	1.706	0.647–4.501	0.281
BMI	1.038	0.963–1.118	0.335
IL-6^max^	1.193	0.952–1.497	0.126

*BMI* body mass index, *CI* confidence interval, *IL-6* interleukin-6, *OR* odds ratio

The upper table represents the percentage of critical COVID-19 patients which require mechanical ventilation during their admission to ICU per biomarker-based cluster. The lower table represents output of the logistic regression analysis which studies the relation between the biomarker-based clusters (presented by dummy variables) and the need for mechanical ventilation in ICU, thereby adjusting for age, gender, body mass index and plasma IL-6 levels. The odds ratios of clusters 1, 3, 4 and 5 over cluster 2 are shown, in addition to the 95% confidence interval of the odds ratio and *p* value of each covariate. *p < 0.05.

**Table 3:** Association between 90-day mortality and biomarker-based clusters in critical COVID-19 patients.

Risk of 90-day mortality in critical COVID-19 patients						
	Clusters	1	2	3	4	5
90-day mortality	yes	2	2	7	11	2
	no	22	21	14	37	2
	Percentage of COVID-19 deceased at 90 days after ICU admission per cluster (%)	8.33	8.7	33.33	22.92	50

Multivariate logistic regression for biomarker-based clusters associated with 90-day mortality			
Covariates	OR	95% CI of OR	*p* value
Intercept	0.005	0.00001–1.446	0.067
Cluster 1-on-2	1.351	0.140–13.045	0.7947
Cluster 3-on-2	5.160	0.819–32.526	0.0806
Cluster 4-on-2	3.069	0.564–16.706	0.1946
Cluster 5-on-2	19.661	1.363–283.571	0.0287 *
Age	1.078	1.010–1.152	0.0241 *
Gender male	2.868	0.742–11.079	0.1266
BMI	0.941	0.853–1.037	0.221
IL-6^max^	0.784	0.596–1.030	0.0802

*BMI* body mass index, *CI* confidence interval, *IL-6* interleukin-6, *OR* odds ratio.

The upper table represents the percentage of critical COVID-19 patients which had deceased at 90-days after ICU admission per biomarker-based cluster. The lower table represents output of the logistic regression analysis which studies the relation between the biomarker-based clusters (presented by dummy variables) and 90-day mortality, thereby adjusting for age, gender, body mass index and plasma IL-6 levels. The odds ratios of clusters 1, 3, 4 and 5 over cluster 2 are shown, in addition to the 95% confidence interval of the odds ratio and *p* value of each covariate. *p < 0.05.

### Relation of individual biomarker trajectories with clinical outcome

**Fig. 6:**
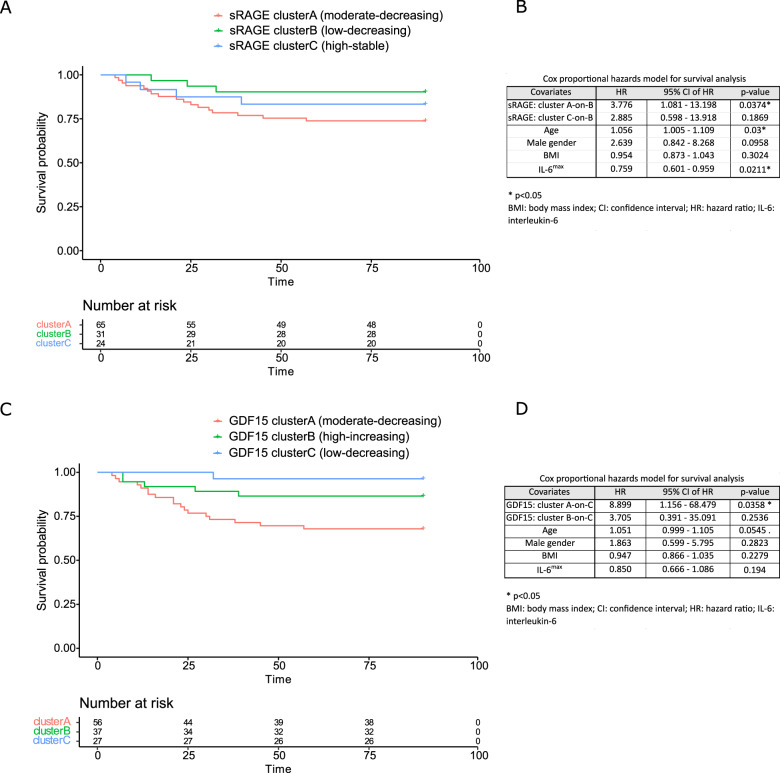
Survival analysis among clusters of critical COVID-19 patients based on trajectories of biomarkers sRAGE and GDF15. **A** Survival curves of the three sRAGE-trajectory-based clusters of critical COVID-19 patients (as defined by longitudinal k-means clustering) were plotted using the Kaplan-Meier method. **B** To assess the effect of the trajectory/kinetics of sRAGE levels in critical COVID-19 patients on the hazard ratio of mortality, Cox proportional hazards modeling was performed with adjustment for age, gender, body mass index and levels of interleukin-6. Patients with sustained moderate sRAGE levels (cluster A) had a 277% higher hazard of mortality compared to patients with a trajectory that started with (and maintained) low sRAGE levels (cluster B), independent from other covariates. **C** Using the Kaplan-Meier method, survival curves of the three GDF15-trajectory-based clusters of critical COVID-19 patients (as defined by longitudinal k-means clustering) were plotted. **D** Cox proportional hazards modeling showed that patients with persistent moderate levels of GDF15 on the three consecutive timepoints (cluster A) have a higher hazard of mortality than those in whom the GDF15 start low and remain low (cluster C), taking into account the covariates age, gender, body mass index and interleukin-6 levels.

All biomarkers were measured in the COVID-19 patients on the first three consecutive days after ICU admission. To examine possible relations of the kinetics of each biomarker with clinical outcomes, we applied machine learning with longitudinal k-means clustering of patients with similar kinetics/ trajectories per individual biomarker. Number of clusters of kinetic trajectories of each individual biomarker ranges from 2 till 6 (Supplementary Fig. S6). This analysis revealed that the risk for mechanical ventilation is higher in patients with either low stable levels of Fe_c_ (cluster B) or moderate/high stable GDF15 levels (clusters A&B) during the first three days in ICU compared to patients with respectively moderate stable levels of Fe_c_ (cluster A) or low stable GDF15 levels (cluster C), after adjusting for age, gender, BMI and IL-6 levels (Supplementary Table S6 and S7). COVID-19 patients with a peak in myoglobin levels at day 2 had more VFDs compared to several clusters with other trajectories of this biomarker (Supplementary Fig. S7). Moderate stable levels of either sRAGE (cluster A) or GDF15 (cluster A) are associated with higher risk for 90-day mortality compared to respectively low stable sRAGE (cluster B) or GDF15 levels (cluster C) (Supplementary Table S8 and S9). This observation is further supported by Kaplan-Meier survival curves showing that patients with moderate stable levels of sRAGE or GDF15 displayed the lowest survival rates, which is also underscored by cox proportional hazards modeling (Fig. 6).

## Discussion

The current study reports on the heterogeneous increase in systemic levels of biomarkers of ferroptosis, iron dysregulation, pyroptosis-related interleukins and pneumocyte cell death in critical COVID-19 patients during the first three days in ICU. Machine learning discerned subgroups with increased pyroptosis-related interleukins, with or without pneumocyte cell death, as well as subgroups with iron dyshomeostasis and ferroptosis. Patients with the highest IL-18 and pneumocyte cell death had the highest SOFA scores and higher risk of mechanical ventilation (but better survival), while the small subgroup with a ferroptotic signature experienced the highest mortality. Kinetics of individual biomarkers, particularly sRAGE and GDF15, over three consecutive measurements are independently associated with mechanical ventilation and mortality.

Firstly, analysis of individual biomarkers revealed that subsets of COVID-19 patients had increases above the reference range of healthy controls. To avoid bias, Gaussian mixture modeling was applied to detect latent subgroups within the cohort of critical COVID-19 patients based on nine biomarkers measured in this study. The first two pyroptosis-related subgroups (each accounting for some 20% of patients) displayed increases in IL-18 with high pneumocyte cell death or high IL-1β without pneumocyte cell death, but had no systemic signature of ferroptosis. While high IL-18 and pneumocyte cell death early in ICU stay increased the risk of mechanical ventilation, it also associates with lower mortality rates. Recently, alveolar epithelial necrosis at an early disease was detected in the early stage of COVID-19-induced ARDS [24]. High IL-1β levels coincided with low pneumocyte cell death and low risk of mechanical ventilation and mortality. Two seminal studies demonstrated that SARS-CoV2-infected circulating monocytes and lung macrophages undergo pyroptosis to halt viral reproduction but release pro-inflammatory triggers during this process [27, 45]. The increase in systemic IL-1β and IL-18 levels is in accordance with other reports in critical COVID-19 patients [46, 47]. One study reported an association between serum levels of IL-18 and mortality and morbidity of hospitalized COVID-19 patients [48]. Our study did not find an increased mortality in the subgroup with high IL-18 in its biomarker profile, indicating that machine-learning-assisted analysis of sets of biomarkers could be advantageous.

Based on the reported increase in pyroptosis-related IL-1β, single inhibition of IL-1 pathway using recombinant IL-1RA anakinra was administered to critical COVID-19 patients without immunological stratification but proved unsuccessful [49]. Bedside machine-learning-assisted biomarker profiling, for instance by means of real-time immunodiagnostics, could have been used to allocate this treatment to the right patients in a personalized medicine approach [46, 50, 51]. Likewise, immunomodulating strategies targeting IL-6 might have benefitted from biochemical stratification [52]. In general the cytokine levels during the so-called cytokine storm from severe COVID-19 are often moderate compared to patients with non-COVID ARDS [53–55]. Direct inflammasome inhibition attenuated COVID-19 severity in a preclinical model, but potent forms of such inhibitors are not yet in clinical use, which leaves the option of inhibiting downstream cytokines IL-1β and IL-18 [56, 57]. To our knowledge, targeted inhibition of IL-18 using recombinant human IL-18 binding protein, called tadekinig alfa, has not been investigated in COVID-19 infections, but it is tempting to hypothesize that this might impact the need for mechanical ventilation [58]. Our group has demonstrated that dual inhibition or deficiency of IL-1 and IL-18 protect against mortality in preclinical models of sepsis and shock, whereas single blocking could not [28]. Such dual cytokine inhibition could be explored in COVID-19 patients.

Secondly, unsupervised clustering identified a subgroup with iron dyshomeostasis (high lactoferrin and Fe_c_) and a smaller subset of critical COVID-19 patients (3.33%) with very high Fe_c_, both of which displayed a tendency towards higher MDA. The subgroup with the highest levels of Fe_c_ (accompanied by high MDA) was independently associated with reduced survival. On the other hand, patients with low stable levels of Fe_c_ on three consecutive measurements also had an adverse outcome, i.e. an increased risk of mechanical ventilation, compared to higher levels. The rise in MDA levels in COVID-19 during ICU admission could be attributed to recurrent ischemia and ischemia-reperfusion damage, loss of control over the unbound iron pool, micro-emboli and a pro-oxidative burst of neutrophils [15–17, 19, 59, 60]. In a preclinical model, SARS-CoV2 was able to induce ferroptosis in the sino-atrial node of the heart [61]. Based on immunohistochemistry a signature of LPO was found in lung parenchyma of patients with non-COVID-19 ARDS [62]. In a Syrian hamster model with SARS-CoV2 infection global redox phospholipidomics revealed increased levels of ferroptotic death signals, i.e. oxygenated phosphatidylethanolamine species among others, in lung tissue and bronchoalveolar lavage fluid [63]. Of note, dexamethasone (frequently used for hospitalised COVID-19) increases the sensitivity for ferroptosis via glutathione depletion [64]. In a previous cohort study of 176 non-COVID ICU patients, we found a cut-off of 2.85 µM for systemic MDA levels early after ICU admission to be predictive for 30-day mortality [21]. Such a cut-off could not be discerned in the current study. We described a clear role for ferroptosis during the multiple organ dysfunction syndrome, but not in preclinical models of sepsis [21]. Likewise, ferroptosis may not act as primordial driver in all critical COVID-19 patients, highlighting the need for patient stratification. We described a myocardial ferroptosis signature in a case of COVID-19-induced myocarditis [65]. It is tempting to suggest that ferroptosis inhibition might impact survival of such patients. Concerning the catalytic iron pool, high Fe_c_ reflects a dysregulation of iron homeostasis and explains the trend towards increased MDA levels in those subgroups. The origin of increased Fe_c_ is unclear, but it might be released from cells with dysregulated autophagy of ferritin, i.e. ferritinophagy [66]. Chakurkar *et al*. demonstrated that higher systemic Fe_c_ and ferritin levels are associated with increased in-hospital mortality in hospitalised COVID-19 patients [67]. Our data suggest that only the highest levels of Fe_c_ levels upon ICU admission bear such prognostic information.

Lastly, analysis of the trajectories of consecutive measurements of sRAGE and GDF15 yielded prognostic information. In this study, moderate stable levels of GDF15 over the first three days in ICU were independently associated with higher mechanical ventilation and lower survival, whereas moderate stable sRAGE levels were an independent predictor of higher mortality. These findings correspond with other studies. Wick et al. reported that hospitalized COVID-19 patients in the highest quartile of sRAGE levels suffered from a lower rate of sustained recovery [68]. Notz et al. reported a similar increase in GDF15 levels during the entire ICU stay of COVID-19 patients [69]. When measured in hospitalized COVID-19 patients GDF15 is independently associated with ICU admission or death [70]. Collectively these results suggest that measurement of sRAGE and GDF15 in the first days of ICU stay could be useful.

This study has some limitations. Firstly, biomarker-based clustering should be replicated in a second independent cohort of critical COVID-19 patients. Moreover, the selection of biomarkers related to certain cell deaths is empirical, but not based on systematic assay, and may be far from fully representative. Indeed, a cluster including 40% of COVID-19 patients had no defining biomarker, indicating that another pathophysiological process may drive their disease progression, such as for instance interferon gamma-induced protein 10 which is reported to be associated with impaired T cell responses in COVID-19 [71]. Other cell death modes such as apoptosis, PANoptosis and NETosis are present in COVID-19 but were not studied in this patient cohort as these were beyond the scope of the current study [72–76]. Lastly, demographics from control groups differed from the critical COVID-19 group, which itself displays a large variation in age for which we adjusted in regression analysis. However, we corrected for gender, BMI and IL-6 levels, which are all reported to have an impact on outcome during severe COVID-19 reducing confounding [46, 77–79].

Collectively, unsupervised clustering points towards a systemic signature of ferroptosis, iron dyshomeostasis, pyroptosis and pneumocyte cell death in subgroups of COVID-19 patients early after ICU admission. This heterogeneity in pathogenetic drivers cannot be discerned by clinical examination alone and impacts clinical outcome in COVID-19, while many cell death modes are druggable. This paves the way towards patient stratification in critical illness to different treatments based on biomarker-profiles, which could be generated bedside in the future [12]. The role of cell death modes, beneficial and/or detrimental and their prognostic importance deserves to be further explored in COVID-19.

## Reporting summary

Further information on research design is available in the Nature Portfolio Reporting Summary linked to this article.

## Supplementary information

Supplementary Information

Reporting Summary

## Data Availability

Data used to support the findings of this study are available from the corresponding author at tom.vandenberghe@uantwerpen.be upon request.
