# Supplementary material for: Ferroptosis and pyroptosis signatures in critical COVID-19 patients
Source: Cell Death Differ. 2023 Aug 15;30(9):2066–77. doi: 10.1038/s41418-023-01204-2 (PMC10482958; doi:10.1038/s41418-023-01204-2)
Supplement: Supplementary file 1 — Supplementary Information [file 41418_2023_1204_MOESM1_ESM.pdf]

**Supplementary Information**

**Ferroptosis and pyroptosis signatures in critical COVID-19 patients**

*Cell Death & Differentiation*

Cédric Peleman, Samya Van Coillie, Symen Ligthart, Sze Men Choi, Jan De Waele, Pieter Depuydt, Dominique Benoit, Hannah Schaubroeck, Sven Marcel Francque, Karolien Dams, Rita Jacobs, Dominique Robert, Ria Roelandt, Ruth Seurinck, Yvan Saeys, Mohan Rajapurkar, Philippe G Jorens, Eric Hoste, Tom Vanden Berghe

Corresponding author: Tom Vanden Berghe, Laboratory of Pathophysiology, Department of Biomedical Sciences, University of Antwerp. Universiteitsplein 1, 2610 Antwerpen, Belgium. tom.vandenberghe@uantwerpen.be, T: +32 3 265 92 50.

## Supplementary methods

*Unsupervised clustering of critical COVID-19 patients into biomarker-based clusters using Gaussian Mixture modelling*

Based on the variation in biomarker levels in COVID-19 patients, we explored whether patients could be separated into clusters based on their profiles of the following biomarkers: MDA, $Fe_c$, ferritin, lactoferrin, myoglobin, IL-1β, IL-18, sRAGE and GDF15. Model-based clustering, more precisely Gaussian Mixture models, was used to visualize clusters with certain volume, shape and orientation, using the mClust package in R version 4.1.1 which employs the expectation-maximization algorithm [1]. This clustering allows for the detection of latent classes based on many independent variables without selection for a dependent variable. In this dataset of critical COVID-19 patients, the 'EEI model' with five diagonal clusters with equal (E) volume, equal (E) shape and the same orientation according to the axes (I) resulted in the lowest Bayesian Information Criterion (BIC) and lowest Integrated Complete-data Likelihood (ICL) [2,3]. The Davies-Bouldin's index was calculated for any given

number of clusters and the elbow method confirmed that separation into 5 clusters provide acceptable separation between clusters [4]. Model-based clustering is frequently used to discern subgroups with distinct profiles of independent variables within heterogenous patient cohorts [5–7].

*Unsupervised clustering of critical COVID-19 patients by trajectories/kinetics of individual biomarkers using longitudinal k-means clustering*

In this study, levels of each biomarker were measured on the first three consecutive days after ICU admission in every critical COVID-19 patient. We aimed to assess the possible relation of kinetics in biomarker levels and clinical disease score in the same time interval, as well as clinical outcome in ICU. Therefore, we constructed trajectories of each biomarker level per individual patient and separated patients in clusters with similar trajectories by longitudinal k-means clustering using the Latrend and Kml packages in R version 4.1.1 [8,9]. The optimal number of clusters for each biomarker was determined by applying the elbow method to find the lowest BIC, lowest Mean absolute error weighted by cluster-assignment probability (WMAE) and highest Model log-likelihood (logLik) in function of the number of clusters. Similar methods were used elsewhere to study the impact of changes in independent parameters on clinical outcomes [10].

All R coding for the unsupervised clustering can be found at: https://github.com/cedricpeleman.

**Supplementary Figure legends**

**Supplementary Figure S1. Comparison of age and body mass index in critical COVID-19 patients, healthy controls and post-operative intensive care unit controls.** (A) Boxplot representing mean, interquartile range and min-max values of age in critical COVID-19 patients (COVID-19), healthy controls (HC) and post-intracranial surgery non-COVID-19 ICU controls (PO). (B) Likewise, body mass index (BMI) is presented for the three groups. Pairwise comparisons of values of biomarkers were performed using the Wilcoxon-rank sum test.

**Supplementary Figure S2. Comparison of interleukin-6 levels in critical COVID-19 patients, healthy controls and post-operative intensive care unit controls.** Boxplot representing log2-transformed values of plasma interleukin-6 (IL-6) myoglobin (the highest value in the first three days after intensive care unit (ICU) admission) in critical COVID-19 patients (COVID-19), as well as single measurements in healthy controls (HC) and post-intracranial surgery non-COVID-19 ICU controls (PO). Pairwise comparisons of values of biomarkers were performed using the Wilcoxon-rank sum test.

**Supplementary Figure S3. Correlogram summarising associations between biomarkers and disease severity scores in the first three days after ICU admission in critical COVID-19 patients without exposure to immunomodulating therapy.** In the subset of critical COVID-19 patients not exposed to immunomodulating therapy (anakinra, siltuximab, tocilizumab) (n=106) we explored associations between the highest value of biomarkers for ferroptosis (i.e. MDA), iron dyshomeostasis (catalytic iron [Fe], ferritin, lactoferrin, myoglobin), pyroptosis-related interleukins (interleukin-1 beta [IL-1β] and interleukin-18 [IL-18]), soluble receptor of advanced glycation end products (sRAGE) and growth differentiation factor 15 (GDF15), as measured systemically within the first three consecutive days after ICU admission. These biomarkers were compared with the highest Sequential organ failure assessment (SOFA) score, APACHE II score (on first day) and lowest P/F ratio in the same time period. The diagonal in the figure displays the distribution of each biomarker in the population as a histogram. Spearman's correlation coefficients between biomarkers and/or clinical scores are presented at the intersection of columns and rows in upper right-hand corner of the figure. Possible non-linear relationships between two biomarkers and/or clinical scores were assessed using locally estimated

scatterplot smoothing (LOESS) regressions on scatterplots at the intersections of rows and columns (presented in lower left-hand corner of the figure). T-tests were performed to assess significance of correlation coefficients; * $p<0.05$, ** $p<0.01$, *** $p<0.001$.

**Supplementary Figure S4. Unsupervised clustering of critical COVID-19 patients based on biomarker profiles by Gaussian Mixture models.** (A) Values of Bayesian Information Criterion (BIC) were plotted for a given number of clusters by each model of Gaussian Mixture modelling. The EEI model with 5 clusters resulted in the lowest BIC value. (B) Likewise, values of the Integrated Complete-data Likelihood (ICL) were plotted per number of clusters by each fitted Gaussian Mixture model. The EEI model with 5 clusters resulted in one of the lowest ICL values. (C) Values of the Davies-Bouldin's index were plotted for any given number of clusters defined by Gaussian Mixture modelling. Lower levels of this index indicate a better separation between the clusters. Increasing the number of clusters to a total of 5 enhanced the separation. (D) Principle component analysis (PCA) plot was used to visualize the 5 clusters of critical COVID-19 patients constructed by the EEI model of Gaussian mixture modelling.

**Supplementary Figure S5. Number of ventilation-free days (VFDs) among different biomarker-based clusters of critical COVID-19 patients.** The number of VFDs was plotted per biomarker-based cluster as discerned by the Gaussian Mixture modelling of the cohort of critical COVID-19 patients (n=120). The possible association of cluster membership with the number of VFDs was explored using multiple linear regression analysis.

**Supplementary Figure S6. Unsupervised clustering of critical COVID-19 patients based on trajectories/kinetics of other individual biomarkers over the first three days after ICU admission.** (A) Longitudinal k-means clustering was used to separate patients into latent subgroups/clusters based on the absolute values and their trajectory/evolution of the biomarker $Fe_c$. Graphs represent the following model parameters in function of the number of clusters: the Bayesian Information Criterion (BIC), the Mean absolute error weighted by cluster-assignment probability (WMAE) and the Model log-likelihood (logLik). The elbow method was used to choose the optimal number of clusters based on the lowest BIC, lowest WMAE and logLik. Concerning the biomarker $Fe_c$, a separation of all critical

COVID-19 patients into 3 clusters was most appropriate. (B) Graphs represent the summarizing trajectory of each cluster of $Fe_c$-based trajectories (in color), while the black lines visualize the kinetic of this biomarker per individual patient that pertains to this cluster. (C) The change in biomarker $Fe_c$ in each of the three constructed clusters is plotted over the first three days after ICU admission. Clusters A and C both start at moderate levels of biomarker $Fe_c$ on the first day of measurement. While the former maintains stable moderate levels, the latter represents patients that develop an increase in $Fe_c$ levels on the next 2 measurements. Cluster B comprises patients that start at low levels of biomarker $Fe_c$ and maintain low levels thereof. (D-K) Critical COVID-19 patients were separated into clusters with distinct trajectories/kinetics by longitudinal k-means clustering for each individual biomarker. Graphs represent the summarizing trajectory of each biomarker in function of time for the following individual biomarkers: MDA, ferritin, lactoferrin and myoglobin, IL-1β, IL-18, sRAGE and GDF15. The optimal number of clusters for each biomarker was determined using the elbow method on BIC, WMAE and logLik parameters (not shown for each individual biomarker).

**Supplementary Figure S7. Relation of the number of ventilation-free days (VFDs) in function of myoglobin-trajectory based clusters of critical COVID-19 patients.** (A) Boxplot represents the mean, interquartile range and min-max values of the number of VFDs in different clusters of critical COVID-19 patients, as defined by unsupervised clustering based on their trajectories of the biomarker myoglobin. (B) Odds ratios (OR), 95% confidence intervals of OR and p-values were generated when comparing the possible association of different myoglobin-trajectory based clusters with the number of VFDs using multiple linear regression. The effect of different clusters was assessed through generation of dummy variables which compared each of the other myoglobin-trajectory-based clusters with the reference cluster F. Regression was performed with adjustment for age, gender, body mass index and interleukin-6 levels. * $p<0.05$, ** $p<0.01$.

**Supplementary Figures**

Supplementary Figure S1

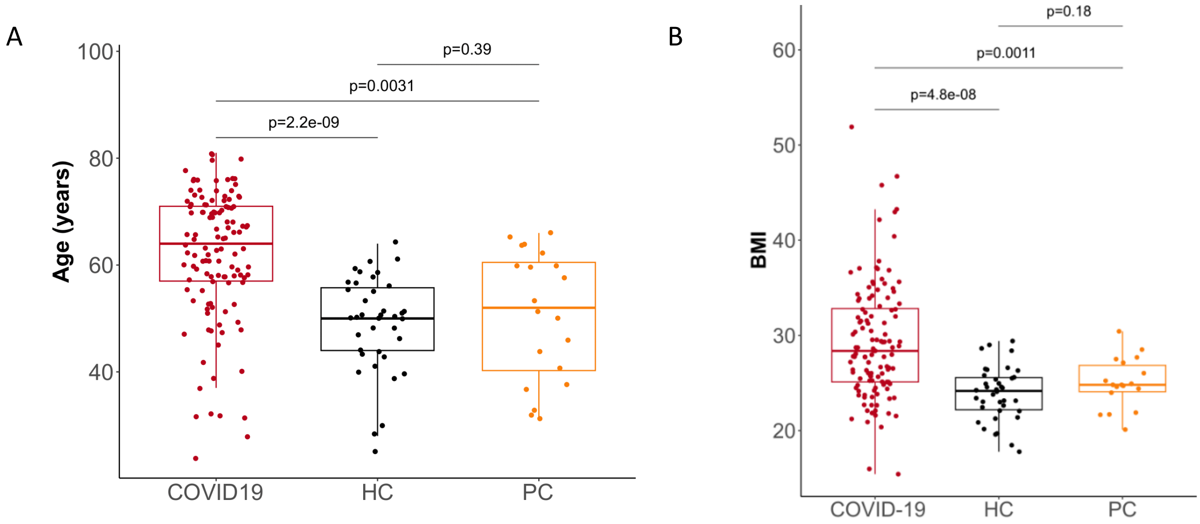

Supplementary Figure S2

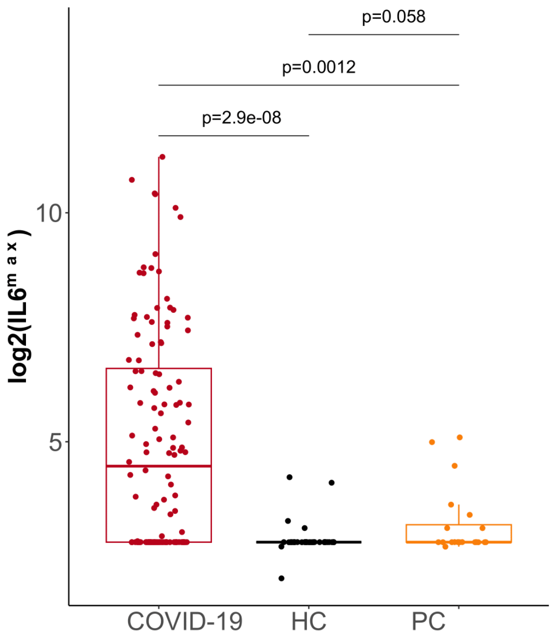

Supplementary Figure S3

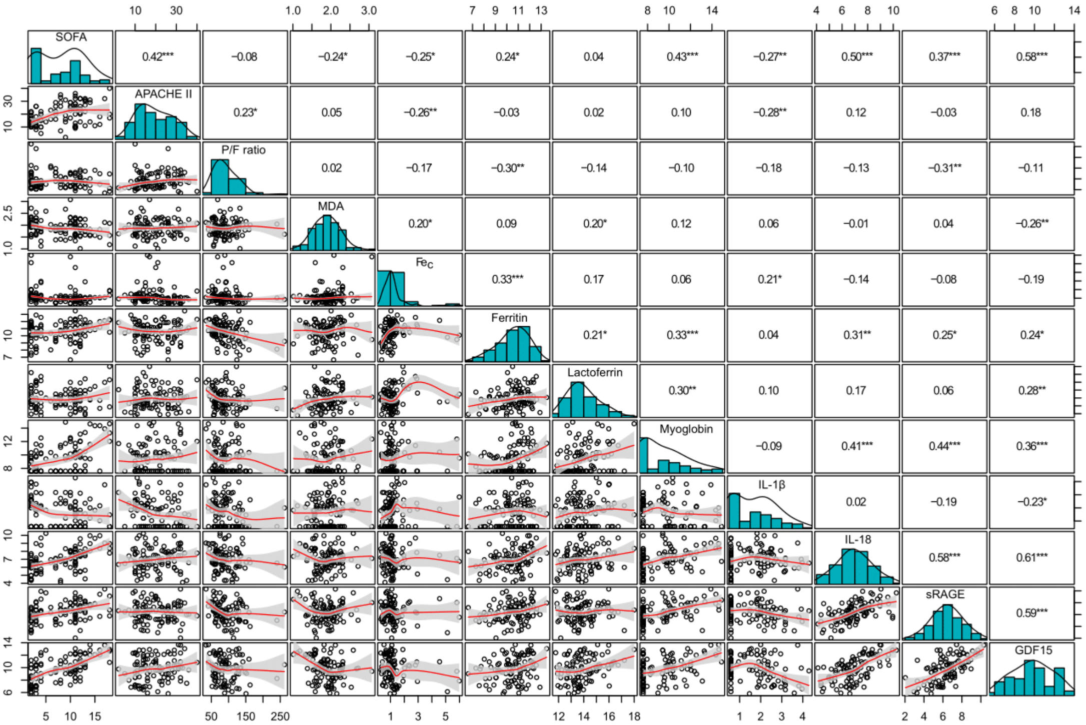

Supplementary Figure S4

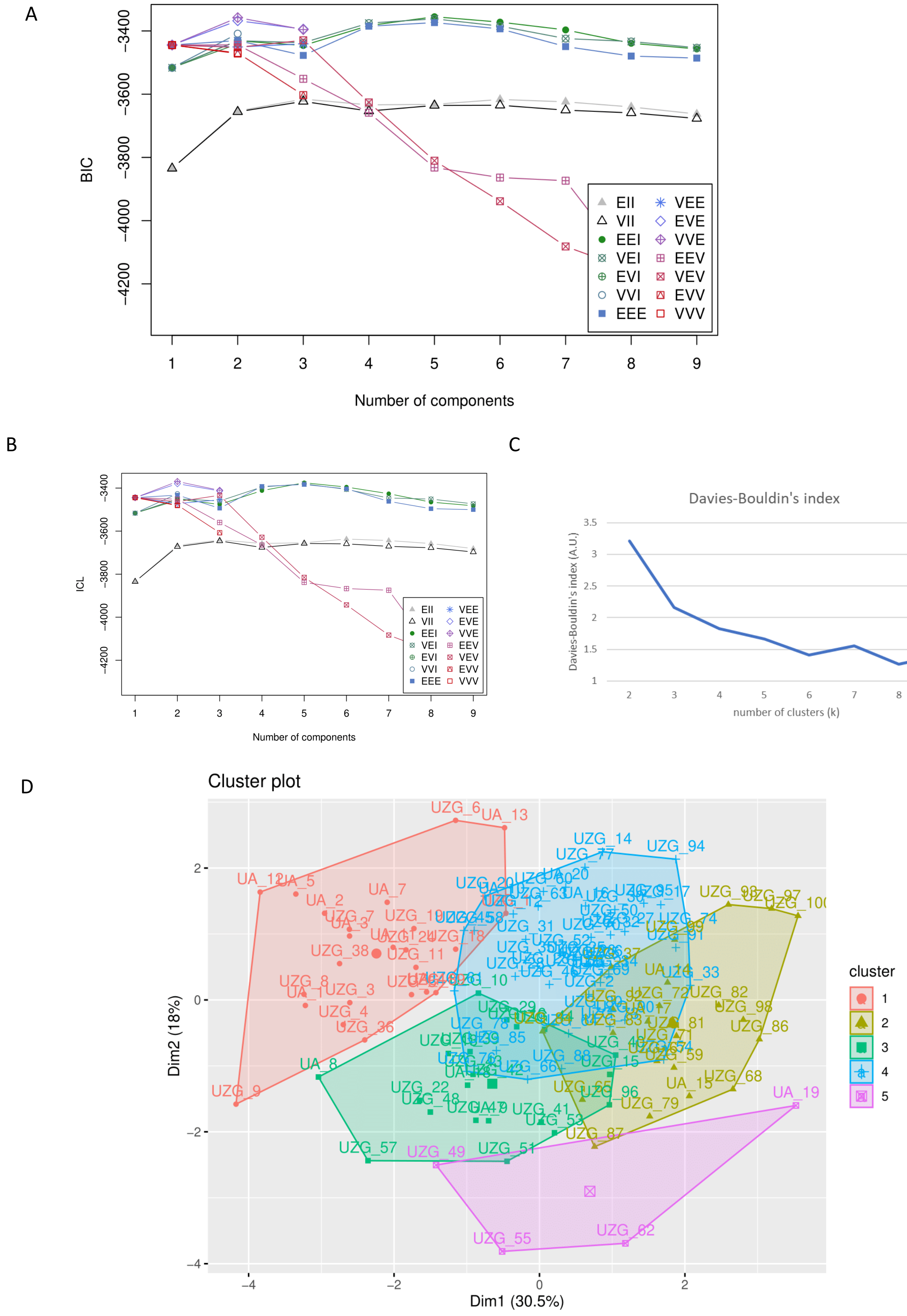

Supplementary Figure S5.

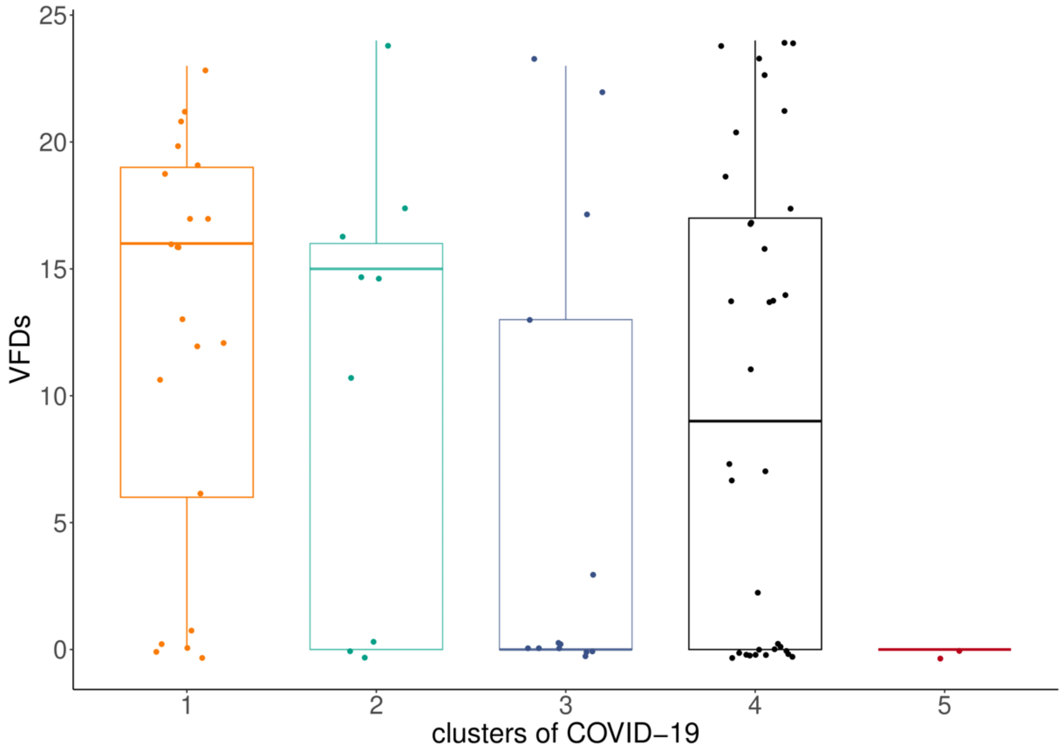

Supplementary Figure S6

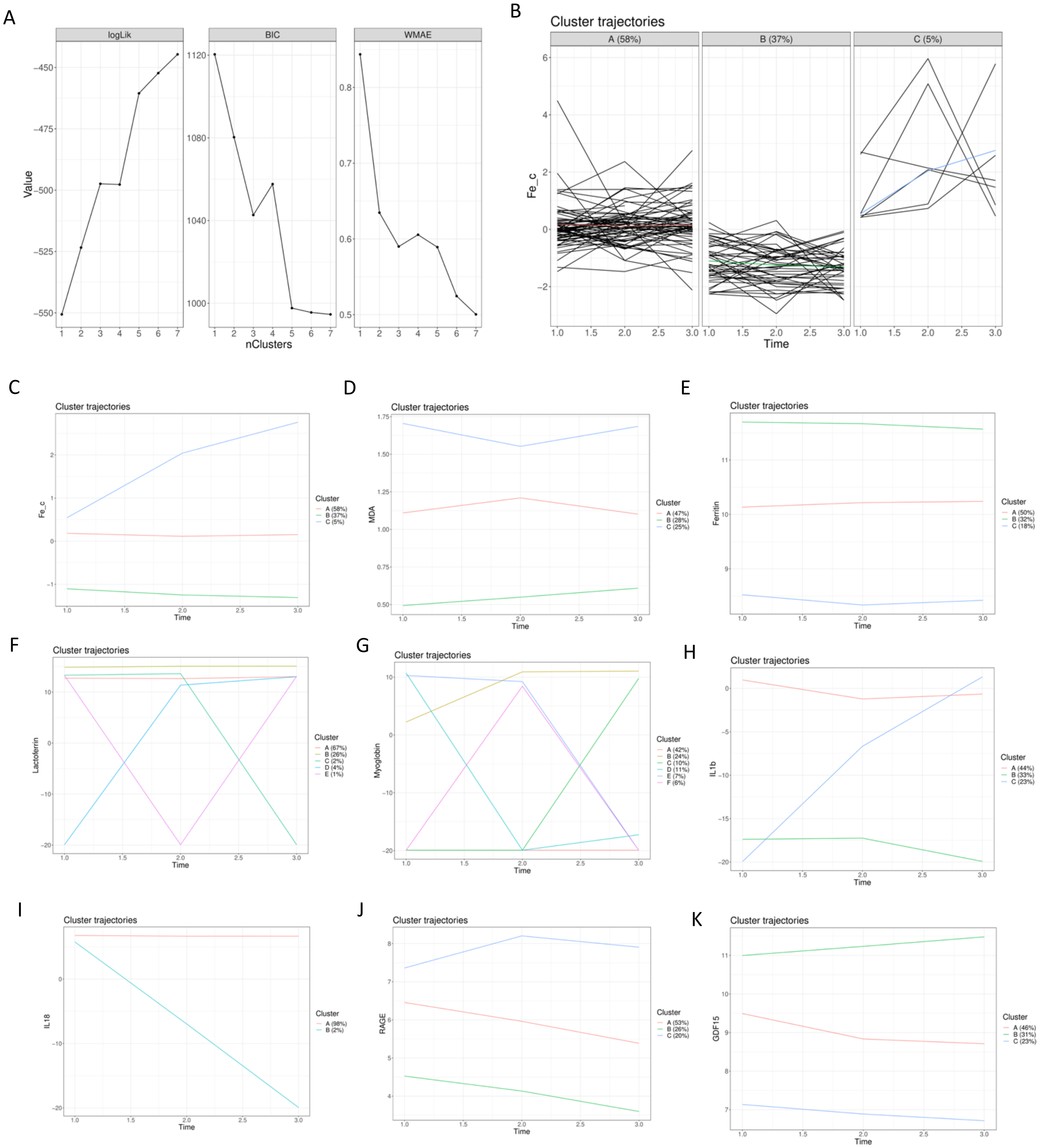

Supplementary Figure S7

A

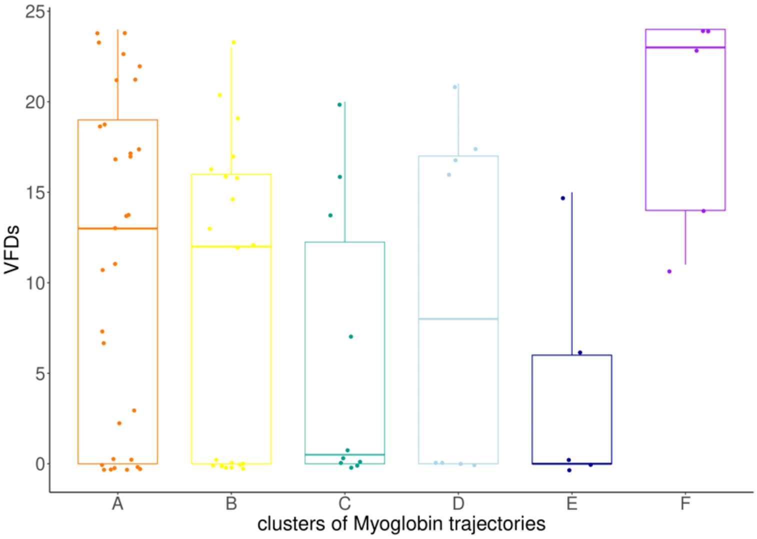


B

Multiple linear regression for Myoglobin trajectories associated with Ventilation free days in ICU

| Covariates | OR | 95% CI of OR | p-value |
|---|---|---|---|
| Intercept | 9.60E+10 | 1223.743 - 7.55E18 | 0.00813 ** |
| Myoglobin: cluster A-on-F | 0.0006 | 2.41E-07 - 1.51 | 0.06765 . |
| Myoglobin: cluster B-on-F | 4.15E-05 | 1.013E-08 - 0.171 | 0.02030 * |
| Myoglobin: cluster C-on-F | 4.56E-07 | 5.52E-11 - 0.00378 | 0.00227 ** |
| Myoglobin: cluster D-on-F | 1.51E-04 | 1.15E-08 - 1.98 | 0.07339 . |
| Myoglobin: cluster E-on-F | 1.80E-07 | 5.84E-12 - 0.006 | 0.00466 ** |
| Age | 0.841 | 0.715 - 0.989 | 0.04032 * |
| Gender male | 0.112 | 0.001 - 11.6 | 0.3581 |
| BMI | 1.04 | 0.76 - 1.42 | 0.80909 |
| $IL\text{-}6^{max}$ | 2.358 | 1.058 - 5.25 | 0.03957 * |

* p<0.05; ** p<0.01

BMI: body mass index; CI: confidence interval; IL-6: interleukin-6; OR: odds ratio

**Supplementary Table legends**

**Supplementary Table S1. Co-morbidities of critically-ill COVID-19 patients and healthy controls.** Data presented as numbers and percentages.

**Supplementary Table S2. Maximum levels of each biomarker during the first three days after ICU admission in critical COVID-19 patients (COVID-19) compared to healthy controls (HC) and post-operative ICU controls (PC).** Data presented as median (IQR).

**Supplementary Table S3. Percentage of patients with increased individual biomarker levels.** Based on the 95% confidence interval of each biomarker in healthy controls (HC) as the reference interval, the percentage of critical COVID-19 patients with increased levels of individuals biomarkers (MDA, $Fe_c$, lactoferrin, myoglobin, IL-1β, IL-18, sRAGE and GDF15) was calculated.

**Supplementary Table S4. Percentage of patients with increases in multiple biomarkers.** The 95% confidence interval of each biomarker in healthy controls (HC) was used as reference interval. The percentage of patients was calculated in whom multiple biomarkers, pertaining to the same pathophysiological process, are elevated above the reference intervals.

**Supplementary Table S5. Biomarker assessment per cluster of critical COVID-19 patients compared to the reference interval in healthy controls.** To assess the magnitude of biomarker elevation in the 5 biomarker-defined clusters of critical COVID-19 patients, the levels were compared to the reference interval (95% confidence interval) of healthy controls. The table provides the percentage of COVID-19 patients with biomarker elevations above the reference interval per cluster, as constructed by Gaussian Mixture modelling.

**Supplementary Table S6. Association between mechanical ventilation in ICU and $Fe_c$ trajectories in critical COVID-19 patients.** The upper table represents the percentage of critical COVID-19 patients which require mechanical ventilation during their admission to ICU per cluster of $Fe_c$ trajectories. The lower table represents output of the logistic regression analysis which studies the relation between the $Fe_c$ clusters (presented by dummy variables) and the need for mechanical ventilation in ICU, thereby

adjusting for age, gender, BMI and plasma IL-6 levels. The odds ratios of $Fe_c$ clusters B and C over cluster A are shown, in addition to the p-value of each covariate. * p<0.05; ** p<0.01.

**Supplementary Table S7. Association between mechanical ventilation in ICU and GDF15 trajectories in critical COVID-19 patients.** The upper table represents the percentage of critical COVID-19 patients which require mechanical ventilation during their admission to ICU per cluster of GDF15 trajectories. The lower table represents output of the logistic regression analysis which studies the relation between the GDF15 clusters (presented by dummy variables) and the need for mechanical ventilation in ICU, thereby adjusting for age, gender, BMI and plasma IL-6 levels. The odds ratios of GDF15 clusters A and B over cluster C are shown, in addition to the p-value of each covariate. ** p<0.01; *** p<0.001.

**Supplementary Table S8. Association between 90-day mortality and sRAGE trajectories in critical COVID-19 patients.** The upper table represents the percentage of critical COVID-19 patients which were deceased at 90-days after ICU admission per cluster of sRAGE trajectories. The lower table represents output of the logistic regression analysis which studies the relation between the sRAGE clusters (presented by dummy variables) and 90-day mortality, thereby adjusting for age, gender, BMI and plasma IL-6 levels. The odds ratios of sRAGE clusters A and C over cluster B are shown, in addition to the p-value of each covariate. * p<0.05.

**Supplementary Table S9. Association between 90-day mortality and GDF15 trajectories in critical COVID-19 patients.** The upper table represents the percentage of critical COVID-19 patients which were deceased at 90-days after ICU admission per cluster of GDF15 trajectories. The lower table represents output of the logistic regression analysis which studies the relation between the GDF15 clusters (presented by dummy variables) and 90-day mortality, thereby adjusting for age, gender, BMI and plasma IL-6 levels. The odds ratios of GDF15 clusters A and B over cluster C are shown, in addition to the p-value of each covariate. * p<0.05.

Supplementary Table S1.

| Co-morbidity, n (%) | COVID-19 (n=120) | Healthy controls (n=39) |
|---|---|---|
| Arterial hypertension | 54 (45) | 3 (23.08) |
| End-stage renal disease | 1 (0.83) | 0 (0) |
| Chronic obstructive pulmonary disease | 8 (6.67) | 0 (0) |
| Diabetes mellitus | 30 (25) | 0 (0) |
| Immune depression | 9 (7.5) | 1 (7.69) |
| Solid-organ transplant | 4 (3.33) | 0 (0) |

Supplementary Table S2.

| Biomarker | COVID-19 (n=120) | HC (n=39) | PC (n=20) |
|---|---|---|---|
| Ferroptosis | | | |
| MDA (µM) | 2.62 (1.17) | 1.63 (0.984) | 0.973 (0.509) |
| Iron dyshomeostasis | | | |
| catalytic iron ($Fe_c$) (µM) | 1.08 (0.72) | 0.54 (0.3) | 0.54 (0.7) |
| lactoferrin (pg/mL) | 14,128 (17,973) | 23,013 (18,353) | 16,174 (24,308) |
| myoglobin (pg/mL) | 338 (1202) | 191 (0) | 191 (1383) |
| Pyroptosis | | | |
| IL1b (pg/mL) | 2.48 (3.96) | 0.5 (2.11) | 0.5 (0.182) |
| IL18 (pg/mL) | 119 (161) | 36.3 (25.7) | 30.6 (31) |
| Cell death and cell damage in general | | | |
| sRAGE (pg/mL) | 91.6 (137) | 45.9 (36.3) | 33 (44.7) |
| GDF15 (pg/mL) | 924 (1677) | 194 (148) | 431 (274) |

Supplementary Table S3.

| log2(biomarker) | 95% CI in HC as reference interval | Percentage of COVID-19 patients above reference interval (%) |
|---|---|---|

| | | |
|---|---|---|
| MDA (µM) | 0.902 - 1.947 | 39.67 |
| $Fe_c$ (µM) | 0.351 - 1.115 | 42.5 |
| Lactoferrin (pg/mL) | 11.588 - 15.640 | 12.5 |
| Myoglobin (pg/mL) | 7.018 - 7.872 | 54.17 |
| IL-1β (pg/mL) | 0.526 - 2.773 | 15.83 |
| IL-18 (pg/mL) | 3.326 - 6.789 | 51.67 |
| sRAGE (pg/mL) | 4.313 - 7.070 | 36.67 |
| GDF15 (pg/mL) | 4.815 - 9.288 | 65.5 |
| HC: healthy controls | | |

Supplementary Table S4.

| Pathophysiological process | Set of biomarkers above reference interval | Percentage of COVID-19 patients above reference interval (%) |
|---|---|---|
| Ferroptosis and iron dyshomeostasis | MDA and $Fe_c$ | 19.17 |
| | $Fe_c$, Lactoferrin, Myoglobin | 5.83 |
| | MDA, $Fe_c$, Lactoferrin, Myoglobin | 2.5 |
| Pyroptosis-related interleukins | IL-1β and IL-18 | 6.67 |
| Alveolar cell death and cell damage in general | sRAGE and GDF15 | 30.83 |

Supplementary Table S5.

| Percentage of COVID-19 patients with biomarker above reference interval of HC per biomarker-based cluster | | | | | |
|---|---|---|---|---|---|
| Clusters | 1 | 2 | 3 | 4 | 5 |
| Percentage with elevated MDA (%) | 20.83 | 43.48 | 52.38 | 37.5 | 75 |
| Percentage with elevated $Fe_c$ (%) | 25 | 56.52 | 90.48 | 18.75 | 100 |
| Percentage with elevated lactoferrin (%) | 8.33 | 4.35 | 57.14 | 0 | 0 |
| Percentage with elevated myoglobin (%) | 79.17 | 43.48 | 52.38 | 45.83 | 75 |
| Percentage with elevated IL-1β (%) | 8.33 | 39.13 | 19.05 | 6.25 | 25 |
| Percentage with elevated IL-18 (%) | 91.67 | 21.74 | 57.14 | 43.75 | 50 |
| Percentage with elevated sRAGE (%) | 100 | 0 | 85.71 | 64.58 | 50 |
| Percentage with elevated GDF15 (%) | 83.33 | 4.35 | 38.1 | 27.08 | 50 |

Supplementary Table S6.

| Need of mechanical ventilation according to $Fe_c$ trajectories in critical COVID-19 patients | | | | |
|---|---|---|---|---|
| | Clusters | A | B | C |
| Need of Mechical Ventilation | yes | 41 | 36 | 2 |
| | no | 27 | 9 | 4 |

| | | | | |
|---|---|---|---|---|
| | Percentage of COVID-19 with need of mechanical ventilation per cluster (%) | 60.29 | 80 | 33.33 |

| Multivariate logistic regression for $Fe_c$ trajectories associated with Mechanical ventilation during ICU stay | | | |
|---|---|---|---|
| Covariates | OR | 95% CI of OR | p-value |
| Intercept | 0.086 | 0.002 - 4.008 | 0.210 |
| Fec: cluster B-on-A | 3.239 | 1.275 - 8.230 | 0.0135 * |
| Fec: cluster C-on-A | 0.585 | 0.094 - 3.641 | 0.566 |
| Age | 1.000 | 0.963 - 1.038 | 0.994 |
| Gender Male | 1.810 | 0.700 - 4.684 | 0.221 |
| BMI | 1.035 | 0.961 - 1.115 | 0.359 |
| $IL\text{-}6^{max}$ | 1.338 | 1.078 - 1.661 | 0.00835 ** |
| | | | |
| * p<0.05; ** p<0.01 | | | |
| BMI: body mass index; CI: confidence interval; IL-6: interleukin-6; OR: odds ratio | | | |

Supplementary Table S7.

| Need of mechanical ventilation according to GDF15 trajectories in critical COVID-19 patients | | | | |
|---|---|---|---|---|
| | Clusters | A | B | C |
| Need of Mechical Ventilation | yes | 41 | 30 | 8 |
| | no | 15 | 6 | 19 |
| | Percentage of COVID-19 with need of mechanical ventilation per cluster (%) | 73.21 | 83.33 | 29.63 |

| Multivariate logistic regression for GDF15 trajectories associated with Mechanical ventilation during ICU stay | | | |
|---|---|---|---|
| Covariates | OR | 95% CI of OR | p-value |
| Intercept | 0.100 | 0.002 - 5.675 | 0.264 |
| GDF15: cluster A-on-C | 6.537 | 2.217 - 19.279 | 0.0007 *** |
| GDF15: cluster B-on-C | 7.283 | 1.928 - 27.516 | 0.003** |
| Age | 0.992 | 0.954 - 1.032 | 0.690 |
| Gender Male | 1.238 | 0.451 - 3.401 | 0.679 |
| BMI | 1.028 | 0.949 - 1.114 | 0.500 |
| IL-$6^{max}$ | 1.263 | 0.995 - 1.604 | 0.054629 . |
| | | | |
| ** p<0.01; *** p<0.001 | | | |
| BMI: body mass index; CI: confidence interval; IL-6: interleukin-6; OR: odds ratio | | | |

Supplementary Table S8.

| 90-day mortality according to sRAGE trajectories in critical COVID-19 patients | | | | |
|---|---|---|---|---|
| | Clusters | A | B | C |
| 90-day mortality | yes | 17 | 3 | 4 |
| | no | 18 | 28 | 20 |
| | Percentage of COVID-19 with 90-day mortality per cluster (%) | 26.15 | 9.68 | 16.67 |

| Multivariate logistic regression for sRAGE trajectories associated with 90-day mortality | | | |
|---|---|---|---|
| Covariates | OR | 95% CI of OR | p-value |
| Intercept | 0.054 | 0.0002 - 13.056 | 0.2969 |
| sRAGE: cluster A-on-B | 0.191 | 0.045 - 0.818 | 0.0257 * |

| | | | |
|---|---|---|---|
| sRAGE: cluster C-on-B | 0.733 | 0.187 - 2.877 | 0.6567 |
| Age | 1.073 | 1.009 - 1.140 | 0.0238 * |
| Gender male | 2.886 | 0.758 - 10.986 | 0.1201 |
| BMI | 0.934 | 0.846 - 1.032 | 0.1791 |
| IL-$6^{max}$ | 0.724 | 0.551 - 0.951 | 0.0204 * |
| | | | |
| * p<0.05 | | | |
| BMI: body mass index; CI: confidence interval; IL-6: interleukin-6; OR: odds ratio | | | |

Supplementary Table S9.

| 90-day mortality according to GDF15 trajectories in critical COVID-19 patients | | | | |
|---|---|---|---|---|
| | Clusters | A | B | C |
| 90-day mortality | yes | 18 | 5 | 1 |
| | no | 38 | 32 | 26 |
| | Percentage of COVID-19 with 90-day mortality per cluster (%) | 32.14 | 13.51 | 3.7 |

| Multivariate logistic regression for GDF15 trajectories associated with 90-day mortality | | | |
|---|---|---|---|
| Covariates | OR | 95% CI of OR | p-value |
| Intercept | 0.007 | 0.00002 - 2.051 | 0.0866 . |
| GDF15: cluster A-on-C | 12.444 | 1.451 - 106.73 | 0.0215 * |
| GDF15: cluster B-on-C | 3.759 | 0.348 - 40.574 | 0.2753 |
| Age | 1.071 | 1.005 - 1.14 | 0.0333 * |
| Gender male | 2.052 | 0.541 - 7.788 | 0.291 |
| BMI | 0.922 | 0.833 - 1.019 | 0.112 |
| IL-$6^{max}$ | 0.829 | 0.63 - 1.091 | 0.180 |

| | | | |
|---|---|---|---|
| | | | |
| * p<0.05 | | | |
| BMI: body mass index; CI: confidence interval; IL-6: interleukin-6; OR: odds ratio | | | |

**Supplementary references**


1 Scrucca L, Fop M, Murphy BT, Raftery AE. mclust 5: Clustering, Classification and Density Estimation Using Gaussian Finite Mixture Models. R J 2016; **8**: 289–317.

2 Neath AA, Cavanaugh JE. The Bayesian information criterion: Background, derivation, and applications. WIREs Comput Stat 2012; **4**: 199–203.

3 Biernacki C, Celeux G, Govaert G. Assessing a Mixture Model for Clustering with the Integrated Completed Likelihood. IEEE Trans Pattern Anal Mach Intell 2000; **22**: 719–725.

4 Davies DL, Bouldin DW. A Cluster Separation Measure. IEEE Trans Pattern Anal Mach Intell 1979; : 224–227.

5 Rosenbaum JT, Choi D, Harrington CA, Wilson DJ, Grossniklaus HE, Sibley CH *et al.* Gene expression profiling and heterogeneity of nonspecific orbital inflammation affecting the lacrimal gland. JAMA Ophthalmol 2017; **135**: 1156–1162.

6 Strigo IA, Simmons AN, Giebler J, Schilling JM, Moeller-Bertram T. Unsupervised learning for prognostic validity in patients with chronic pain in transdisciplinary pain care. Sci Rep 2023; **13**: 7581.

7 Bornschein J, Wernisch L, Secrier M, Miremadi A, Perner J, MacRae S *et al.* Transcriptomic profiling reveals three molecular phenotypes of adenocarcinoma at the gastroesophageal junction. Int J Cancer 2019; **145**: 3389–3401.

8 Den Teuling N. Package ‘latrend’ version 1.5.1: a Framework for Clustering Longitudinal Data. 2023; : 1–171.

9 Genolini C, Falissard B. Kml: A package to cluster longitudinal data. Comput Methods Programs Biomed 2011; **104**: e112–e121.

10 Allen NB, Siddique J, Wilkins JT, Shay C, Lewis CE, Goff DC *et al.* Blood pressure trajectories in early adulthood and subclinical atherosclerosis in middle age. Jama 2014; **311**: 490–497.
